# Measuring quality and level of care provided by family caregivers of persons with dementia: protocol for a systematic review of validated instruments

**DOI:** 10.3934/publichealth.2021016

**Published:** 2021-03-01

**Authors:** Afeez Abiola Hazzan, Joyce O Hazzan, Mark Oremus

**Affiliations:** 1 Department of Healthcare Studies, the College at Brockport, State University of New York, 350 New Campus Drive, Brockport, New York, 14420, USA; 2 School of Business, Liberty University, 1971 University Blvd., Lynchburg, VA, 24515, USA; 3 School of Public Health and Health Systems, University of Waterloo, 200 University Avenue West, Waterloo, ON, N2L3G1, Canada

**Keywords:** aging, family caregiving, dementia, Alzheimer's disease, quality of care, level of care, population health

## Abstract

**Background:**

Changes in the quality-of-life (QoL) of family caregivers as they care for their loved ones with dementia over time may affect the quality or level of care that these caregivers provide. We do not know if validated instruments exist to directly measure this quality or level of care. The purpose of this systematic review is to identify validated instruments for measuring the quality or level of care provided by family caregivers of persons with dementia.

**Methods:**

We will include articles containing a validated instrument or questionnaire designed to measure quality or level of care provided by the aforementioned group of caregivers. The included articles may utilize the tools in primary or secondary data analyses, or evaluate the tools' psychometric properties. The following electronic databases will be searched from inception date to the present: Medline, CINAHL Complete, Cochrane Central, and PsycINFO. We will also search the gray literature, the reference lists of included articles, and contact experts in the field to help identify relevant instruments. Included articles will be required to report on an instrument measuring at least one of the following outcomes: quality of care, level of care, appropriateness of care, amount of time spent providing care, and caregiver performance. Two reviewers will independently screen retrieved citations, extract data, and assess the methodological quality of each included article. A narrative synthesis method will be used to describe the findings.

**Discussion:**

Results of this systematic review will show whether validated instruments exist to measure the quality or level of care provided by family caregivers of persons living with dementia. This will make it possible to develop initiatives that are targeted towards improving the quality or level of care provided by family caregivers.

## 1. Background

Primary informal caregivers are mainly responsible for the care of persons with dementia (PwD), including Alzheimer's disease (AD). Also known as “family caregivers”, these caregivers are relatives, friends, or neighbors who provide unpaid assistance to PwD [1],[2]. This assistance involves a variety of tasks, including shopping for groceries, helping with medications, managing finances, and overseeing legal affairs [2]–[5]. Research has shown that caregiving becomes more demanding as the disease progresses over time; as such, the quality-of-life (QoL) experienced by dementia caregivers is generally lower than the QoL of individuals who care for persons with other diseases [3]–[7]. Since the average person lives approximately 10 years following a dementia diagnosis, changes in the QoL of family caregivers over time might affect the quality or level of care provided to PwD [3]–[7].

In a published systematic review examining the relationship between caregiver QoL and the quality of care provided, only one study was found to be somewhat relevant [5]. However, this single study did not specifically examine the association between QoL and quality or level of care in PwD. The review's authors concluded that the absence of research was due to the lack of instruments or questionnaires designed to measure level or quality of care in dementia [5].

Measuring the quality or level of care provided by dementia caregivers might facilitate the identification of potential barriers to QoL for both the family caregiver and PwD. This will be particularly useful to stakeholders (e.g. policymakers, care providers) if the barriers to QoL could be ameliorated through interventions or policies. For example, this could facilitate an investigation into how changes in family caregiver QoL during a pandemic like COVID-19 may affect the ability of family caregivers to provide optimum quality or level of care to PwD. Indeed, many family caregivers face increased challenges during COVID-19 including social isolation, unavailability of in-home health services, inadequate respite care, and lack of informal support [8],[9]. However, such research could not be conducted without a validated instrument to measure quality or level of care provided by family caregivers of PwD. Further, we do not know if any such validated instruments currently exist. Therefore, the objectives of the proposed systematic review are:

1. To investigate whether validated instruments or questionnaires are available for measuring the quality and/or level of care provided by family caregivers of older adults living with dementia in community settings.

2. To investigate the psychometric properties of the identified instruments or questionnaires.

## 2. Methods

The systematic review described in this protocol will follow the PRISMA statement [10],[11]. Further, the PRISMA-P checklist (see Appendix A) was followed in writing this systematic review protocol [12],[13]. In addition, this systematic review protocol has been registered with PROSPERO (registration number: CRD42021224702). This protocol will serve as a plan for the systematic review process [12],[13].

### 2.1. Population

This systematic review will focus on instruments or questionnaires designed for completion by unpaid family caregivers of PwD.

### 2.2. Concepts examined

The purpose of the systematic review is to identify validated instruments or questionnaires designed to measure quality or level of care provided by family caregivers of persons living with any type of dementia (for example, Alzheimers disease, vascular dementia, dementia with lewy bodies, frontotemporal lobar dementia). In addition, we will report the psychometric properties of any instruments or questionnaires identified in the literature search.

Quality or level of care can be defined broadly, depending on the specific context. Some studies have used questionnaires or instruments that measure related concepts (or proxy measures) of quality or level of care provided by family caregivers of PwD [7],[14]–[16]. Some proxy measures for quality of care include: caregiver mastery (i.e. Caregiver Mastery Index) and task management strategy (i.e. Task Management Strategy Index). Further, proxy measures for level of care include: caregiver willingness to provide care, as well as the amount of time spent providing care. Therefore, the current systematic review will include studies that have used questionnaires or instruments measuring these and other relevant proxy measures of quality or level of care provided by family caregivers of PwD. For example, quality of care could be operationalized using any means (caregiver mastery, task management strategy, and so on) that have been designed to measure this concept [7],[14]. Further, level of care includes caregiver willingness to provide care and the amount of time spent providing care [15],[16].

### 2.3. Information sources and literature search

The following electronic databases will be searched from inception to the present: Medline, CINAHL Complete, Cochrane Central, and PsycINFO. We will also perform internet searches to identify gray literature, hand search the reference lists of included articles, and contact experts in dementia family caregiving research to identify additional relevant instruments.

### 2.4. General search terms for electronic databases

The following terms will be used to formulate search strategies for the electronic databases: older adults or elderly; quality of care; appropriateness of care; level of care; amount of time spent providing care; caregiver performance; caregiver (unpaid or family or informal); measuring instrument; questionnaire; scale; validated; community settings; dementia or Alzheimers disease or vascular dementia or dementia with lewy bodies or frontotemporal lobar dementia.

A sample search syntax for Medline was developed by a professional librarian (see Appendix B). The syntax will be updated for each database and the internet search. Also, the search strategy/syntax will be customized for each database. Results from the literature search will be uploaded to Covidence, an online software that will be used for screening. We will extract data into an Excel spreadsheet.

### 2.5. Study eligibility criteria

We will include peer-reviewed studies containing instruments or questionnaires that measure the quality or level of care provided by primary informal caregivers of PwD. Only instruments or questionnaires used in the caregiver population will be considered. Included studies must report at least one of the following outcomes in relation to the instrument or questionnaire: quality of care, level of care, appropriateness of care, amount of time spent providing care, caregiver performance, or similar variables pertaining to primary informal caregiving roles. We will include studies that have used these instruments as outcome measures of the quality or level of care provided by family caregivers of PwD. We will include studies published in any country and written in the English language.

Due to the ambiguity involved in defining what constitutes “quality” or “level” of care [17],[18], studies where the authors have described using instruments for measuring these constructs will be eligible for inclusion in the review. This encompasses studies measuring “quality” or “level” of care from a task-oriented perspective.

### 2.6. Study selection and data collection process

Two reviewers will independently screen the titles and abstracts of studies identified in the literature search. Studies meeting the eligibility criteria after title and abstract screening will advance to full-text screening. A detailed data collection form will be developed to tabulate information from the included articles (for example, how the instrument was developed, its intended use, psychometric properties, year it was developed, country where it was developed). Data will be extracted using a form which will be piloted by two reviewers and further refined if necessary. Reviewers will meet to resolve discrepancies by consensus. In cases where studies report the use of a relevant instrument over different time periods, data will be extracted from each time period. Further, in situations where multiple publications report data from an instrument that was found to be relevant to the current study, the different uses of the instrument across these papers will be reported. When data are not clearly reported or when the appropriateness of the instrument is in doubt, the lead author of the study will be contacted for clarification.

### 2.7. Assessment of risk of bias

The risk of bias of each included study will be assessed at both the outcome level (i.e. focusing on quality or level of care) and the study level in order to provide a robust estimation of the bias associated with each included study. If a study utilizes a randomized controlled trial design, the Cochrane Risk of Bias Tool will be used for assessing risk of bias in the study [19]. The Cochrane Effective Practice and Organisation of Care Risk of Bias Tool will be used for assessment of risk of bias for controlled clinical trials, interrupted time series, and controlled before-after studies utilizing the identified measuring instruments [20],[21]. Further, the Newcastle-Ottawa Scale will be used for studies employing cohort and case-control designs [22]. In addition, we will use the COSMIN checklist to assess the methodological quality of studies that focus on the measurement properties of the identified instruments or questionnaires [23],[24].

### 2.8. Evidence synthesis

A narrative synthesis method will be used to describe the results of this systematic review and summary tables will be created to show the key characteristics of each instrument. If identified instruments are found to be too heterogeneous, then separate qualitative analyses will be presented for each instrument identified, and graphical representation may be used to display the main study findings.

## 3. Discussion

We intend for the results of this systematic review to show whether validated instruments exist to measure the quality or level of care provided by family caregivers of PwD. In addition, the specific psychometric properties (including validity and reliability) of any identified instruments will be reported. Investigating whether instruments exist to directly measure quality or level of care provided by family caregivers and then evaluating the psychometric properties of such instruments could be a good starting point for further research on this topic, including developing and validating an instrument. The results could provide stakeholders including support organizations, government agencies, and policy-makers with information about tools that can help them make informed decisions about initiatives to optimize the quality or level of care provided to PwD.

The findings of this systematic review will be published in an open-access peer-reviewed journal to make the results widely available to family caregivers, clinicians, researchers, and policy-makers. Also, the results will be presented at relevant national and international research meetings.
